# Structure of native glycolipoprotein filaments in honeybee royal jelly

**DOI:** 10.1038/s41467-020-20135-x

**Published:** 2020-12-08

**Authors:** Simone Mattei, Arvid Ban, Armin Picenoni, Marc Leibundgut, Rudi Glockshuber, Daniel Boehringer

**Affiliations:** 1 Department of Biology, Institute of Molecular Biology and Biophysics, ETH Zurich, Otto-Stern-Weg 5, Zurich, 8093 Switzerland; 2 Present Address: Imaging Centre, European Molecular Biology Laboratory, Meyerhofstraße 1, 69117 Heidelberg, Germany; 3 Present Address: Cryo-EM Knowledge Hub, ETH Zurich, Otto-Stern-Weg 3, Zurich, 8093 Switzerland

**Keywords:** Structural biology, Supramolecular assembly, Cryoelectron microscopy

## Abstract

Royal jelly (RJ) is produced by honeybees (*Apis mellifera*) as nutrition during larval development. The high viscosity of RJ originates from high concentrations of long lipoprotein filaments that include the glycosylated major royal jelly protein 1 (MRJP1), the small protein apisimin and insect lipids. Using cryo-electron microscopy we reveal the architecture and the composition of RJ filaments, in which the MRJP1 forms the outer shell of the assembly, surrounding stacked apisimin tetramers harbouring tightly packed lipids in the centre. The structural data rationalize the pH-dependent disassembly of RJ filaments in the gut of the larvae.

## Abstract (web summary)

Female honeybee larvae develop into queens when they are fed exclusively with royal jelly (RJ) and major royal jelly protein 1 (MRJP1) is the main protein component of RJ. Here, the authors present the 3.5 Å cryo-EM structure of native RJ filament, where MRJP1 forms the outer shell surrounding apisimin tetramers with bound lipids at the core of the filament.

## Introduction

Royal jelly (RJ) is a gelatinous, protein-, carbohydrate-, and lipid-rich secretion produced by the mandibular glands of nurse honeybees (*Apis mellifera*)^1^. In honeybee colonies, female castes are nutritionally regulated and only larvae exclusively fed on RJ develop into queens^1–3^. The major royal jelly glycoproteins in RJ are involved in cast determination of female larvae but the underlying mechanisms are still not fully understood^4–8^. The major royal jelly protein 1 (MRJP1), a member of the family of yellow proteins with a molecular mass of ~60 kDa, is the most abundant glycoprotein in RJ^1,9^. The RJ glycoproteins are secretory proteins of the hypopharyngeal glands^10^. At pH values below 4.5, as found in native RJ upon acidification in the mandibular glands^11^, MRJP1 assembles with the small (5 kDa) RJ protein apisimin into long, hetero-polymeric filaments that can reach lengths above 1 µm^12^. These filaments were recently shown to be responsible for the high viscosity of RJ, guaranteeing that RJ-embedded queen bee larvae remain stably attached to the ceiling of the vertically oriented queen bee cells^12^. The presently available structural information on MRJP1 is based on a crystal structure determined at neutral pH, in which the MRJP1 was found in a planar, tetrameric complex together with four molecules of apisimin and eight molecules of 24-methylenecholesterol^13^, consistent with the reported oligomeric state of MRJP1 in solution at neutral pH^14^. However, the architecture and composition of the MRJP1-containing filaments that form at the native acidic pH 4.0 of RJ^11^ still remained unknown. Here we present the structure of native RJ filaments using a combination of cryogenic electron microscopy (cryo-EM) approaches based on tomography and helical reconstruction methods. The obtained reconstruction allowed us to define the composition and architecture of the native RJ filaments and to rationalize spontaneous RJ filament association at low pH and later dissociation at elevated pH values in the larvae gut.

## Results

### Purification of native RJ filaments

We purified RJ filaments from RJ by means of ammonium sulfate precipitation and size exclusion chromatography (SEC) at pH 4.0 (see methods). SDS-PAGE and mass spectrometry analysis confirmed that MRJP1 was the most abundant protein component of the filaments (Supplementary Fig. 1), which eluted at a SEC volume corresponding to a molecular mass above 500 kDa. In addition, Edman sequencing confirmed the presence of equimolar amounts of MRJP1 and apisimin in the RJ filament preparation. For our structural analysis we proceeded using the protein material from high-molecular mass SEC fractions containing the RJ filaments.

### Cryo-electron tomography and subtomogram averaging reveal the architecture of RJ filaments

Purified RJ filaments were vitrified and imaged to acquire 11 dose-symmetric tilt series (Supplementary Fig. 2). From the reconstructed tomograms we could identify 241 RJ filaments. An ab initio structure of the native filaments was determined by a reference-free, iterative alignment and averaging procedure of three-dimensional particles (subtomograms) that were extracted from one individual filament. The resulting low-resolution ab initio reconstruction showed the overall architecture of the purified RJ filaments consisting of an H-shaped unit oligomerizing into a symmetric helical assembly by stacking interactions (Supplementary Fig. 3). The available crystal structure of the D2 symmetric MRJP1_4_/apisimin_4_/24-methylenecholesterol_8_ oligomer could be fitted with confidence as rigid body within one H-shaped unit. The RJ helical assembly retains the D2 point group symmetry of the tetramers since both of the 2-fold rotational symmetry axes are orthogonal to the longitudinal axis of the filaments. Therefore, the RJ helical assembly has no polarity.

The alignment of the full dataset of 4045 subtomograms with applied D2 symmetry yielded a final reconstruction of the native RJ filament at 8 Å resolution with clearly discernible secondary structure elements (Supplementary Fig. 3). By rigid-body fitting the crystallographic model of the crystallized RJ filament building block^13^ into the calculated subtomogram averaging map we were able to reveal the architecture of the native RJ filaments that consist of symmetric MRJP1_4_/apisimin_4_/24-methylenecholesterol_8_ units that are stacked to form a helical assembly with 54 Å rise and 64° rotation (Supplementary Fig. 3). Although the RJ helical assembly is overall linear and does not present supercoiling, the filaments observed in our tomograms show noticeable flexibility (Supplementary Fig. 2). This conformational landscape is also manifested as a considerable variation of the local resolution along the longitudinal axis of our subtomogram averaging map (Supplementary Fig. 3).

### The high-resolution real-space helical reconstruction shows *N*-glycans and lipids within the RJ filament

**Fig. 1:**
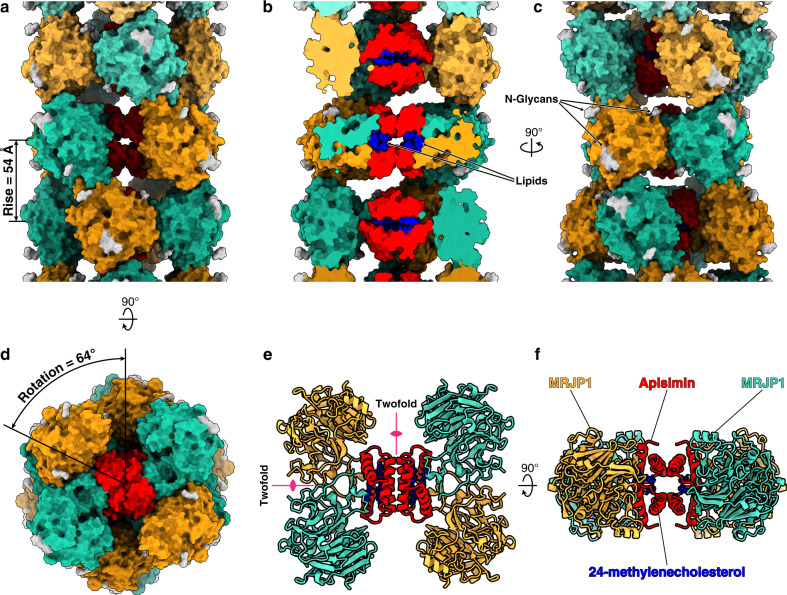
Architectural overview of the native RJ filaments. **a**–**d** The native RJ helical assembly is shown as surface representation. **a** Side view, the helical rise is shown. **b** Cross section along the longitudinal axis. **c** As in **a** after 90° rotation along the longitudinal axis. **d** Top view, the helical rotation is shown. The MRJP1 subunits in each MRJP_4_-apimisin_4_ hetero-octamer layer are alternately colored in orange and cyan to distinguish subunits from different layers. Apisimin is colored in red, 24-methylenecholesterol in blue, and N-glycans in silver. **e**, **f** The H-shaped MRJP1_4_/apisimin_4_/24-methylenecholesterol_8_ complex is shown from two orthogonal orientations with proteins represented as ribbons and the lipids as sticks. **e** Top view with the two 2-fold symmetry axes indicated as pink lines. **f** Side view along one of the 2-fold axes.

To determine the structure at high resolution we used real-space helical reconstruction methods starting from accurate estimates of the helical rise and rotation angle determined from our subtomogram averaging reconstruction (Fig. 1a, d and Supplementary Fig. 4). The resulting map, calculated at 3.5 Å resolution (Supplementary Figs. 4 and 5), was used for rebuilding the structure starting from the previous crystal structure and including the peripheral MRJP1 protein loops responsible for filament-forming inter-subunit contacts (Fig. 1 and Supplementary Figs. 6 and 7). Overall, we did not observe major conformational changes of MRPJ1 and apisimin in the RJ filaments with respect to the crystal structure determined at neutral pH.

The MRJP1 primary structure contains three putative post-translational *N*-glycosylation sites with the consensus sequences Asn-X-Ser or Asn-X-Thr. The glycosylation at these sites was suggested based on experiments that revealed a molecular mass shift from 57 kDa to 47 kDa after treatment of MRJP1 with *N*-glycosidase F^15^, and based on the crystal structure of MRJP1–apisimin where electron density was observed for a *N*-acetyl-β-D-glucosamine (NAG) residue at Asn144, even after de-glycosylation by peptide:*N*-glycanase F. Our high-resolution map of the native MRJP1 filament shows clear densities branching from all three asparagine residues (Asn28, Asn144, and Asn177) predicted to have *N*-linked glycans (Supplementary Fig. 6). Although the intrinsic flexibility of the *N*-linked glycans limits the map resolution at those sites, we were able to model one NAG residue at Asn28, two NAG residues at Asn144, and two NAG residues plus a β-D-mannose (BMA) residue at Asn177 (Fig. 1c). Our structure thus provides structural evidence for these post-translational modifications at all three predicted sites.

In addition to the N-linked glycans, we observed two unassigned, elongated densities at the core of the MRJP1_4_/apisimin_4_/24-methylenecholesterol_8_ oligomers (Supplementary Fig. 7a, b). The densities are ~25 Å long and are located at the center of the oligomer within a highly hydrophobic environment formed by the apisimin alpha helix 2 and the outer 24-methylenecholesterol from each asymmetric unit of the tetrameric oligomer. Although building an atomic model for this region was not possible, the highly hydrophobic chemical environment and the shape and length of the observed densities are consistent with a fatty acid with aliphatic tails of 12–16 carbons, or possibly a mixture of those. Lipids are abundant in RJ, accounting for 3–6% of its wet weight^16–19^ and the release of these fatty acids in the midgut of the honeybee larvae following the digestion of the RJ filaments has been suggested to play a role in the cast determination induced by the RJ diet^20,21^. The stoichiometric binding of lipids might play a role in the biogenesis of the MRJP1_4_/apisimin_4_/24-methylenecholesterol_8_ filament units by stabilizing the interface between the apisimin subunits similar to the role of stoichiometrically bound lipids for assembly of F family pili^22^.

### Mechanism of RJ filament assembly at acidic pH

**Fig. 2:**
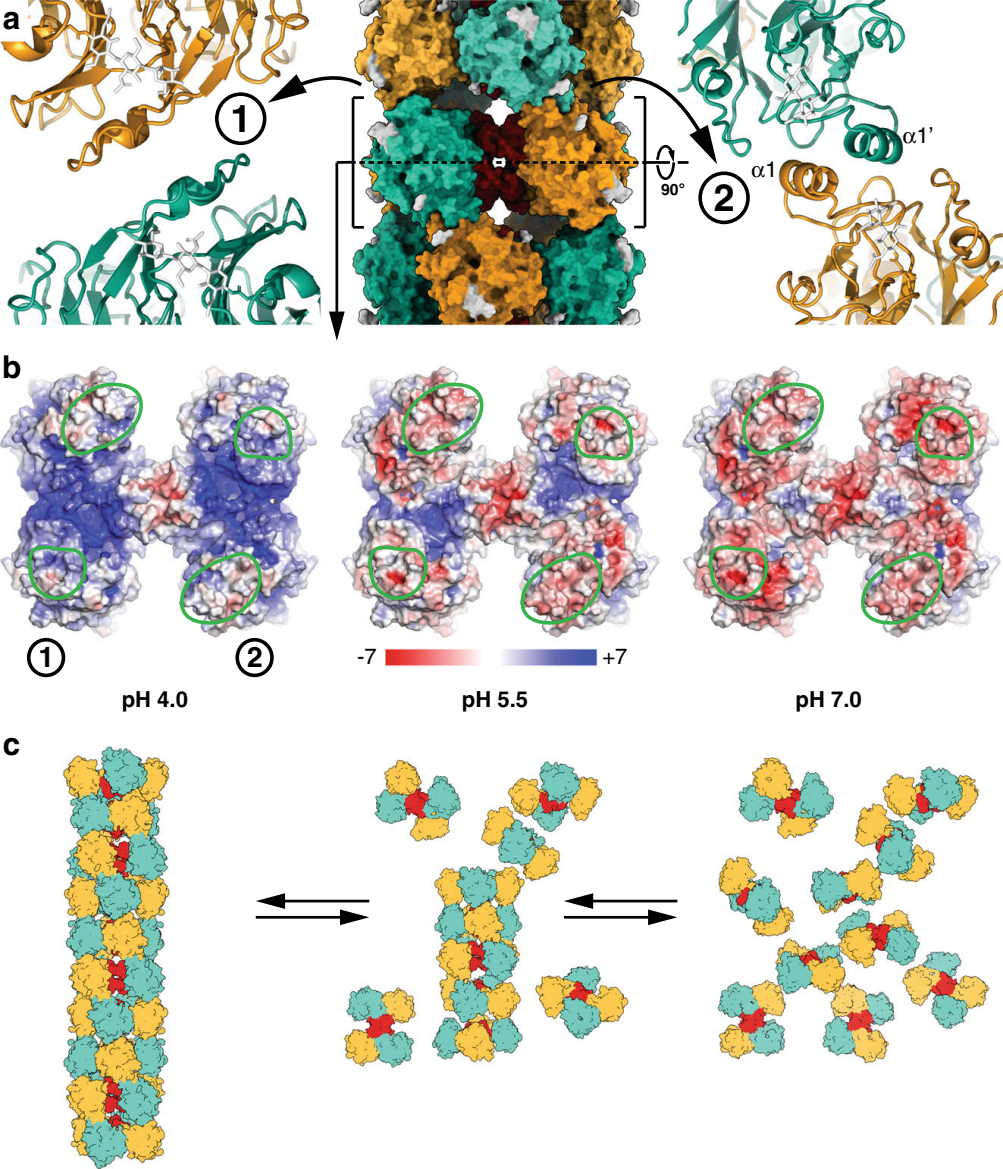
The pH-dependent disassembly of RJ filaments. **a** At pH 4.0, two major interfaces (labeled 1 and 2) between symmetry-related hetero-octamers exist in the RJ filament. Subunits (shown as surface in the overview and cartoons in the close-ups) are colored as in Fig. 1. The major contact (label 2) is mediated by homodimerization of MRJP1 alpha helices 1 (α1). **b** Top views of the electrostatic surface potentials (± 7 kT/e) of one hetero-octameric layer calculated at different pH values (see Methods for details). At pH 4.0, the four contact areas (1606 Å^2^ in total) between the hetero-octamer layers (encircled in green) are predominantly hydrophobic and allow stable assembly of the hetero-octamers into RJ filaments. At the higher pH values observed in the gut of bee larvae, the interaction sites become negatively charged. The repulsion between the hetero-octamer layers leads to destabilization and eventually disassembly of the RJ filament. **c** Schematics showing the pH-dependent assembly model of RJ filaments.

The stacked MRJP1_4_/apisimin_4_/24-methylenecholesterol_8_ building blocks are the basic RJ filament units that interact via two major interfaces involving a small number of electrostatic and hydrophobic interactions of MRJP1 (Fig. 2a, b). One of the contacts is mediated by homodimerization of MRJP1 alpha helices 1 (α1, residues 47–56) from neighboring building blocks that mainly interact via hydrophobic interactions involving Gln51, Ile54, and Leu55 of helix 1 as well as Tyr61 in the following loop (Supplementary Fig. 7c). At the *N*-terminus of helix 1, Glu48 is located in close proximity to Phe395 and Asp396 of the neighboring RJ filament unit (Supplementary Fig. 7c). The helical assembly is further stabilized by a homodimerization interface present on the opposite side of the MRJP1 protein (Fig. 2a, b), involving homodimerization of a short turn (residues 280–281) followed by a loop segment (residues 287–295) that includes His294 and Asp292. This loop is partially disordered in the previously published crystal structure determined at neutral pH, suggesting that it becomes ordered upon filament formation at acidic pH.

Oligomerization of the RJ units into filaments is probably dependent on the protonation state of the glutamate residues (pKa ~ 4.3)^23^, which are mostly protonated at pH 4.0. Upon exposure of MRJP1 to lower pH in the mandibular glands where RJ is produced, the electrostatic repulsion between the RJ filament units is lowered, allowing them to assemble into filaments via mostly hydrophobic contacts (Fig. 2b, c). RJ filament polymerization may proceed by addition of a new filament unit to either of the two ends of a growing filament. The loss of repulsive negative charges is the reason why close contacts that would be prohibited at neutral pH are observed between acidic residues, including Glu48 and Asp396. Additional stabilization of the filament at low pH likely arises from charge–charge interactions, including the interaction between His294 that is positively charged at low pH and Asp292 of MRJP1 on the neighboring RJ filament unit that would partially be negatively charged at pH 4.0. Although there is no sequence or structural similarity between any RJ proteins and spider silk proteins, the RJ filament formation is conceptually reminiscent to the self-assembly of spider silk proteins that occurs during secretion upon reduction of pH and involves protonation of glutamate residues^24–26^. Once RJ filaments are ingested by the bee larvae, the high pH found in the honeybee midgut will induce the disassembly of RJ filaments, destabilization of the MRJP1 protein^27^, and lead to its proteolytic degradation by digestive enzymes.

In conclusion, our structural analysis provides a comprehensive description of the architecture of the glycolipoprotein RJ filaments, and suggests a role of electrostatic interactions in their pH-dependent assembly upon secretion from the mandibular glands of the honeybee and later disassembly in the midgut of the honeybee larvae.

## Methods

### RJ filaments purification

RJ filaments were purified from commercially available RJ (Bio-Gelée Royale (100% pure, 15% protein content), Naturwaren-Niederrhein GmbH, D-47574 Goch-Asperden, Germany). Briefly, a sample with an estimated 8 mg/ml total RJ protein concentration was prepared by dissolving 2.67 g of RJ in 50 ml of water. The sample was dialyzed overnight at 4 °C using a 3.5 kDa molecular weight cut-off membrane against 5 l of 10 mM formic acid-NaOH pH 4.0. The total protein was isolated by ammonium sulfate precipitation by adding 39 g of solid (NH_4_)_2_SO_4_ to 64 ml of dialyzed RJ solution. The sample was incubated at 4 °C for 2 h under light stirring. Precipitated RJ proteins were pelleted by centrifugation in a Sorvall SS34 rotor at 47,850×*g* for 60 min. The supernatant was discarded and the protein pellet was dissolved in 21 ml of 10 mM formic acid-NaOH pH 4.0. The dissolved RJ protein sample was dialyzed against 5 l of the same buffer for 4 h to remove the remaining ammonium sulfate and then filtered through a 0.2 µm pore filter prior to gel filtration. 60 mg of total protein were separated on a Superdex 200 26/60 gel filtration column (320 ml volume) equilibrated with 10 mM formic acid-NaOH pH 4.0, 150 mM NaCl. Eluted proteins were detected via their absorbance at 280 nm, and fractions of 5 ml were collected. The fractions just outside of the exclusion volume (C1–C4) were pooled for the structural analysis of the high-molecular mass RJ filaments (Supplementary Fig. 1a). According to molecular mass standards for calibration of the same gel filtration column, the pooled fractions corresponded to a molecular mass above 500 kDa.

### Edman sequencing

The presence of apisimin and the molar MRJP1/apimisin ratio in purified RJ filaments was measured by quantitative Edman sequencing (Alphalyse A/S, Odense, Denmark) after calibration of HPLC peak intensities with an equimolar mixture of the 20 natural amino acids. Six consecutive degradation cycles confirmed the six *N*-terminal residues of MRJP1 (NILRGE) and apisimin (KTSISV). The two residues identified in each degradation step always proved to be the most abundant degradation products. The average molar ratio between the respective MRJP1 and apisimin residue in these six degradation steps was 1.15 ± 0.32, confirming the 1:1 ratio between MRJP1 and apisimin in the RJ filament structure within experimental error.

### SDS-PAGE and mass spectrometry (MS)

The protein content of samples collected after each step of protein purification was analyzed by SDS-PAGE and MS. The protein samples were mixed with denaturing and reducing sample buffer and heated at 95 °C for 10 min prior to loading. The samples were then loaded on a polyacrylamide gel consisting of a stacking portion (4% acrylamide, 0.1% SDS, and 0.125 M Tris-HCl buffer pH 6.8) layered on top of the resolving portion (15% acrylamide, 0.1% SDS, and 0.4 M Tris-HCl buffer pH 8.8).

The gel was then stained with Coomassie blue to visualize the protein bands. In order to determine the identity of individual proteins in the fractions, protein bands were cut from the gel and sent for protein identification by MALDI-TOF mass spectrometry of tryptic peptides. Briefly, gel bands were cut into small pieces and washed twice with 100 µl of 100 mM NH_4_HCO_3_/50% acetonitrile, then washed once with 50 µl of acetonitrile. The supernatant was discarded and the bands were incubated at 60 °C for 34 min with 10 µl of trypsin solution (5 ng/µl in 10 mM Tris-HCl/2 mM CaCl_2_, pH 8.2) and 30 µl buffer (10 mM Tris-HCl/2 mM CaCl_2_, pH 8.2). After incubation, the supernatant was removed and the gel pieces extracted with 150 µl of 0.1% TFA/50% acetonitrile for 15 min by ultrasonic treatment. Samples were dried and dissolved in 20 µl of 0.1% trifluoroacetic acid (TFA). 1 µl of each sample was mixed 1:1 with MALDI matrix solution (1.4 mg/ml HCCA in 85% acetonitrile, 0.1% TFA, 1 mM NH_4_H_2_PO_4_) and spotted on the target. Database searches were performed by using the Mascot (Swiss-Prot, TrEMBL) search programs. The main protein band was confirmed to be MRJP1, the other two prominent bands running above the MRJP1 band and below the MRJP1 band in the total protein lane labeled RJ (Supplementary Fig. 1b) were determined to be MRJP3 and MRJP2, respectively.

### Cryo-EM sample preparation

A solution of 0.2 mg/ml graphene oxide was applied to glow discharged Quantifoil R2/2 grids and incubated for 90 s. The excess of graphene oxide solution was removed by paper blotting and the grids were air-dried. 5 µl of sample solution from gel filtration fractions C1–C4 (Supplementary Fig. 1a) were applied to each graphene oxide-coated grid and plunge-frozen using a Vitrobot Mark IV (Thermo Fisher Scientific) operated at 12 °C with 95% humidity.

### Cryo-EM data acquisition – tomography

Micrographs for electron cryo-tomography were acquired using a Titan Krios transmission electron microscope (Thermo Fisher Scientific) operated at 300 KeV, equipped with a Quantum post-column energy filter and a K2 direct electron detector (Gatan). Automated data acquisition was controlled using SerialEM^28^. A total of 11 tilt series were acquired at a nominal magnification of 105,000 x resulting in a calibrated pixel size of 1.387 Å. Tilt series were acquired using a dose-symmetric Hagen-scheme^29^ with a 3° tilt increment and an angular range of ± 60° and nominal defocus between −4.0 and −6.3 µm. Each tilt image was exposed for 1.6 s for a total flux of 2.2 e^-^/Å^2^, resulting in a total flux of 90 e^-^/Å^2^ for a full tilt series of 41 micrographs. Each tilt image was acquired as a movie of four frames.

### Cryo-EM data processing – tomography

#### Tomography

Movie frames of the tilt-series were aligned and averaged using of the MotionCor2 software^30^. Contrast transfer function (CTF) estimation of each motion-corrected tilt image was performed using CTFFIND4^31^. Images were low-pass filtered according to the cumulative dose based on the previously published critical exposure curve^32^. Tilt series alignment and tomogram reconstruction were performed using IMOD^33^. Subsequent extraction, alignment, and averaging of three-dimensional particles (subtomograms) was performed using the Dynamo software package^34^. From the reconstructed tomograms, 241 RJ filaments were identified and a total of 12,416 subtomograms were extracted along the spline of the filaments using a 3-fold oversampling with respect to the distance of the filament layers observable from the tomogram. An ab initio structure of the native RJ filaments was determined by a reference-free, iterative alignment and averaging procedure of 211 motion-corrected, dose-filtered, and CTF-corrected subtomograms that were extracted from one individual filament with a pixel size of 11.1 Å and a box size of 48 pixels. The average of the extracted subtomograms was used as initial reference. The in-plane angular search range was 360° with a 30° step while the out-of-plane cone search range was 30° with a 15° step. Throughout the iterative alignment procedure, the subtomograms were aligned against a reference low-pass filtered at 40 Å, and all search angles were refined twice from the found local minima with final search steps of 7.5° and 3.75° for the in-plane search and the out-of-plane search, respectively. The obtained reference was then re-centered and re-oriented within its box to have the longitudinal axis of the filament aligned with the *z*-axis and intersecting the center of the box. Given the observed lack of filament polarity, the 12,416 subtomograms extracted from all filaments were then pooled together and iteratively aligned as described above. After alignment convergence, subtomograms that converged on overlapping positions (distance cutoff of 4 pixels corresponding to 44.4 Å) were removed. For each position only the subtomogram with the best cross-correlation score was retained. The remaining 4045 subtomograms were then re-extracted with a voxel size of 5.55 Å using a box size of 64 pixels and iteratively aligned and averaged. The initial in-plane and out-of-plane cone search ranges were 20° with a 10° step. Throughout the iterative alignment procedure, the subtomograms were aligned against a reference low-pass filtered at 40 Å and all search angles were refined four times from the found local minima with final search step of 1.25° for both the in-plane search and the out-of-plane search. The subtomograms were then extracted with a voxel size of 2.77 Å using a box size of 96 pixels and split in two half-datasets that were further processed independently as “odd” and “even” datasets for gold standard procedure and aligned with applied D2 symmetry. The initial in-plane and out-of-plane cone search ranges were 10° with a 5° step. The subtomograms were aligned against a reference low-pass filtered at 17 Å and all search angles were refined four times from the local minima with final search steps of 0.625° for both the in-plane search and the out-of-plane search. The calculated FSC curve calculated by masking the central hetero-octameric subunit resulted in a final reconstruction of the native RJ filament at 8.8 Å resolution using the FSC = 0.143 criterion.

### Cryo-EM data acquisition – helical reconstruction

Micrographs for helical reconstruction were acquired using a Titan Krios transmission electron microscope (Thermo Fisher Scientific) operated at 300 KeV, equipped with a Quantum post-column energy filter and a K3 direct electron detector (Gatan). A total of 6656 micrographs were automatically collected using the SerialEM software^28^. For each micrograph a stack of 34 images was recorded in super-resolution mode at a nominal magnification of 105,000 x, corresponding to a calibrated pixel size of 0.42 Å/pixel, with defocus ranging between −1 and −3 µm. The total exposure time was 1.7 s, resulting in a total electron dose of ~82 e^−^/Å^2^. Data acquisition parameters are summarized in Table S1.

### Cryo-EM data processing – helical reconstruction

Movie frames acquired for helical reconstruction were motion-corrected, dose-weighted, and Fourier-cropped to a final pixel size of 0.84 Å using MotionCor2^30^. CTF parameters of non-dose-weighted and motion-corrected micrographs were estimated using Gctf^35^. Motion-corrected micrographs were visually inspected to assess the quality of their power spectra and the distribution of RJ filaments, and 1990 micrographs were selected for further processing. Subsequent data processing was carried out using the implementation of the Iterative Helical Real Space Reconstruction (IHRSR) approach to helical processing within Relion 3.0^36^. Filaments were manually picked from 76 micrographs and 7004 particles were extracted with a box size of 512 pixels and rescaled by Fourier-cropping resulting in a final pixel size of 3.36 Å and box size of 128 pixels. Initial 2D classification was performed to obtain class averages of the picked filaments. Four class averages were selected and used as templates to automatically pick 406,638 particles from the full dataset. The picked particles were extracted with a box size of 512 pixels applying a binning factor of 4, resulting in a final pixel size of 3.36 Å /pixel and a box size of 128 pixels. One round of 2D classification was used to remove featureless particles, and 270,434 particles were selected for further processing. 3D classification of the selected particles was performed applying the helical parameters obtained from the tomographic reconstruction and providing a featureless cylindrical density with a height of 430 Å and an outer diameter of 370 Å as reference. One class showing structural features comparable to the helical assembly obtained by subtomogram averaging was selected for further processing. The 240,483 particles assigned to the selected class were re-extracted with a box size of 512 pixels and rescaled by Fourier-cropping resulting in a final pixel size of 1.19 Å and a box size of 360 pixels. The extracted particles were aligned using 3D refinement and submitted to a skip-alignment 3D classification focused on 8 MRJP1 subunits for further removal of misaligned particles. The refinement and post-processing of the 43,130 selected particles yielded a map resolved at 3.9 Å resolution. After per-particle CTF refinement the structure was refined and resolved at 3.5 Å resolution. (See Supplementary Fig. 4 for the processing scheme and Supplementary Fig. 5 for the quality of the cryo-EM map).

### Model building and refinement

For atomic interpretation of the 3.5 Å cryo-EM map (Supplementary Fig. 5), one MRJP_1_/apisimin_1_ protomer of the published crystal structure (PDB 5yyl)^13^ was docked into the central, best-resolved area of the map using UCSF ChimeraX^37^ and adjusted in COOT^38^. Unassigned areas of density, including non-modeled side chains, missing protein loops and residues at the *N*-terminus of MRJP1, a sulfate ion, and glycosylation sites were built manually. The final model of the protomer included residues 20–431 of MRJP1 and residues 33–76 of apisimin. After symmetry expansion to a model comprising four hetero-octameric MRJP_4_/apisimin_4_ layers, the coordinates were subjected to five cycles of real space refinement using PHENIX^39^, during which protein secondary structure, side chain rotamer, and Ramachandran restraints as well as strict NCS between protomers were applied (Table S1). For PDB deposition, the two central hetero-octameric layers were kept and re-refined for an additional cycle to update the B factors and model statistics, while the two peripheral layers located in less well-ordered areas of EM density were removed. The geometry of the final model was validated using MolProbity. The real space correlation coefficient (CC_mask_) between the cryo-EM map and the refined model was 0.83, indicating an excellent fit, and the model-versus-map FSC at the FSC = 0.5 criterion resulted in a similar resolution as the one calculated from the map half-sets at the FSC = 0.143 criterion (Supplementary Fig. 5E).

### Surface potential calculation

The surface potentials shown in Fig. 2 were calculated with the PDB2PQR server^40,41^ using the AMBER forcefield^42^ in conjunction with PROPKA^43–45^ to assign the protonation state at the provided pHs. The surface potentials were visualized in PyMOL (The PyMOL Molecular Graphics System, Version 2.0 Schrödinger, LCC) using the APBS^41^ plugin with a color gradient from −7 kT/e (red) to +7 kT/e (blue). The calculations were performed using one entire hetero-octameric layer, while cofactors and glycosyl moieties were excluded. For histidines built in alternative conformations, only one of the rotamers was included at full occupancy.

### Reporting summary

Further information on research design is available in the Nature Research Reporting Summary linked to this article.

## Supplementary information

Supplementary Information

Reporting summary

## Data Availability

Cryo-EM maps and atomic models have been deposited in the Electron Microscopy Data Bank (EMDB) and wwPDB, respectively, with the following accession code EMD-11892 and PDB 7ASD (RJ filament real space helical reconstruction); EMD-11898 (tomographic reconstruction of RJ filaments). Other data are available from the corresponding authors upon reasonable request.
