# Supplementary material for: Structure of native glycolipoprotein filaments in honeybee royal jelly
Source: Nat Commun. 2020 Dec 8;11:6267. doi: 10.1038/s41467-020-20135-x (PMC7722742; doi:10.1038/s41467-020-20135-x)
Supplement: Supplementary file 1 — Supplementary Information [file 41467_2020_20135_MOESM1_ESM.pdf]

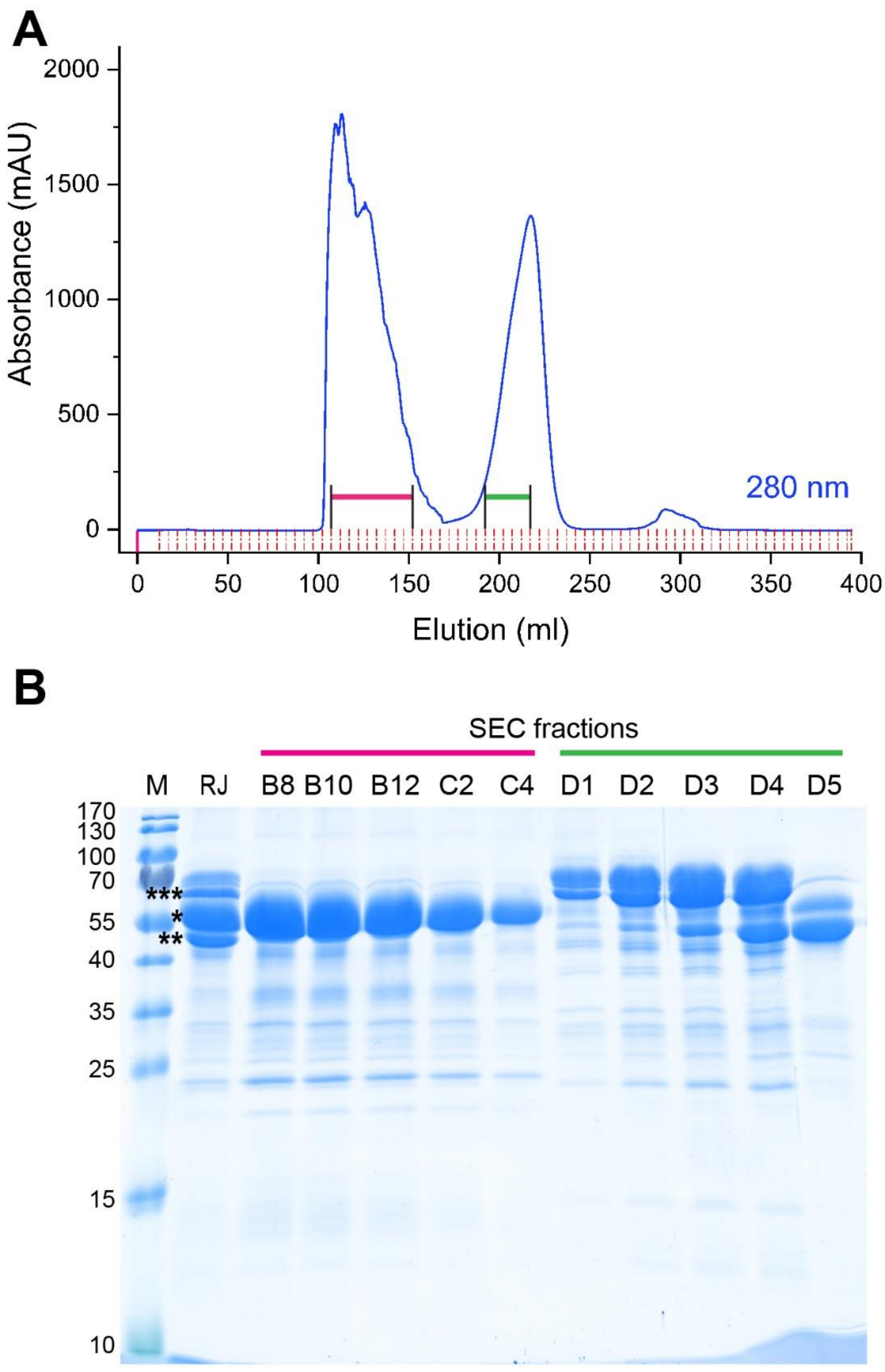

Supplementary Figure 1. Purification of native RJ filaments. (**A**) Absorbance at 280 nm of the eluted proteins after size exclusion chromatography (SEC) of the purified RJ sample separated on a Superdex 200 26/60 gel filtration column. The red marks on the elution axis define the 5 mL collected fractions, the magenta and green bars highlight the fractions analysed by SDS-PAGE. (**B**) The purified RJ protein sample used as SEC input (RJ) and the SEC fractions (B8-C4 and D1-D5) collected for protein analysis visualized by Coomassie-stained SDS-PAGE. M: Molecular mass standard. Themain protein bands in (RJ) are MRJP1 (*****, major protein component in SEC fractions B8-C4), MRJP2 (******, major protein component in SEC fraction D5), MRJP3 (*******, major protein component in SEC fractions D1-D4).

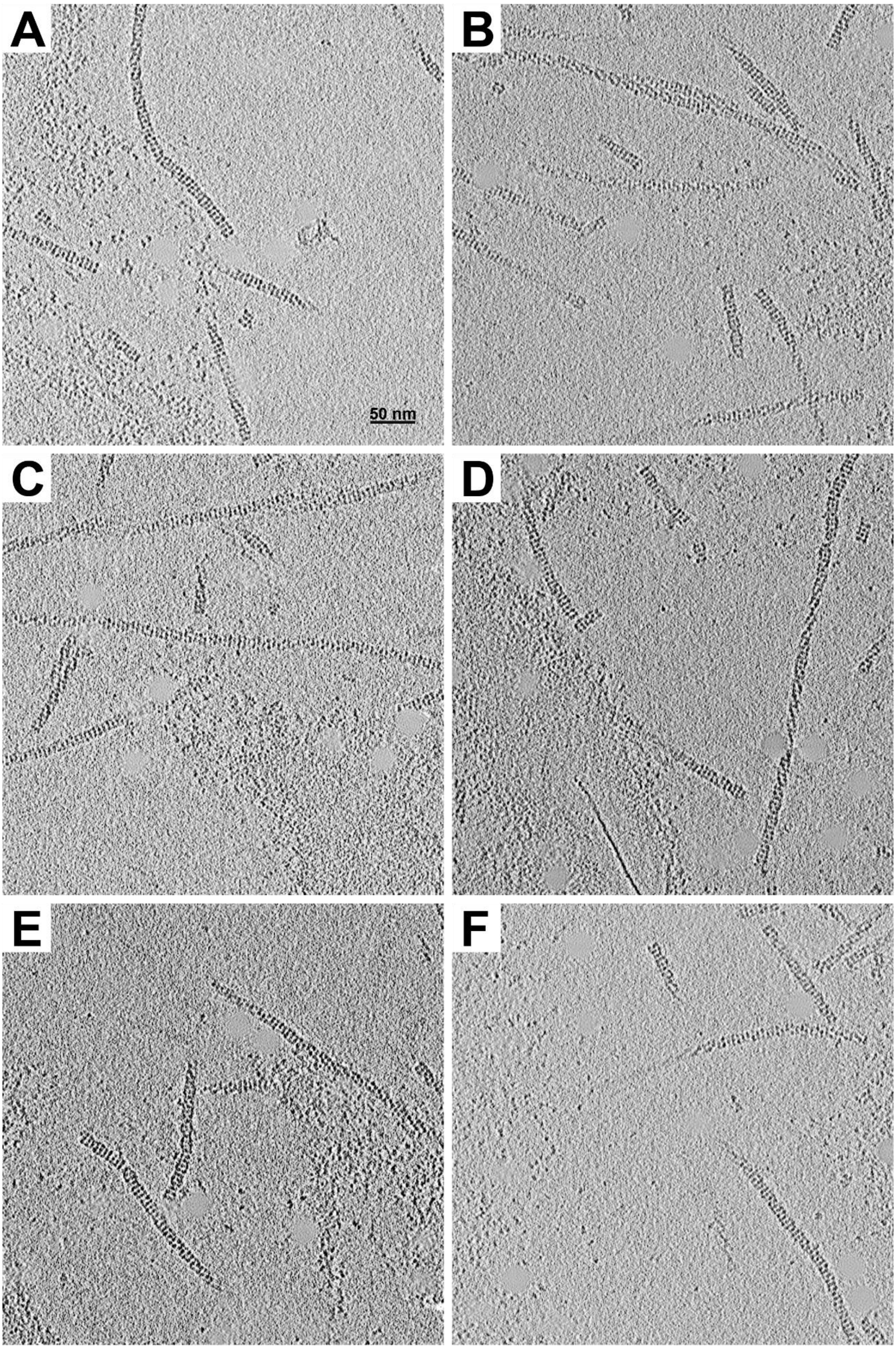

Supplementary Figure 2. Tomographic reconstructions of native RJ filaments. (**A-F**) Computational slices through six representative tomographic reconstructions of native RJ filaments. Scale bar 50 nm.

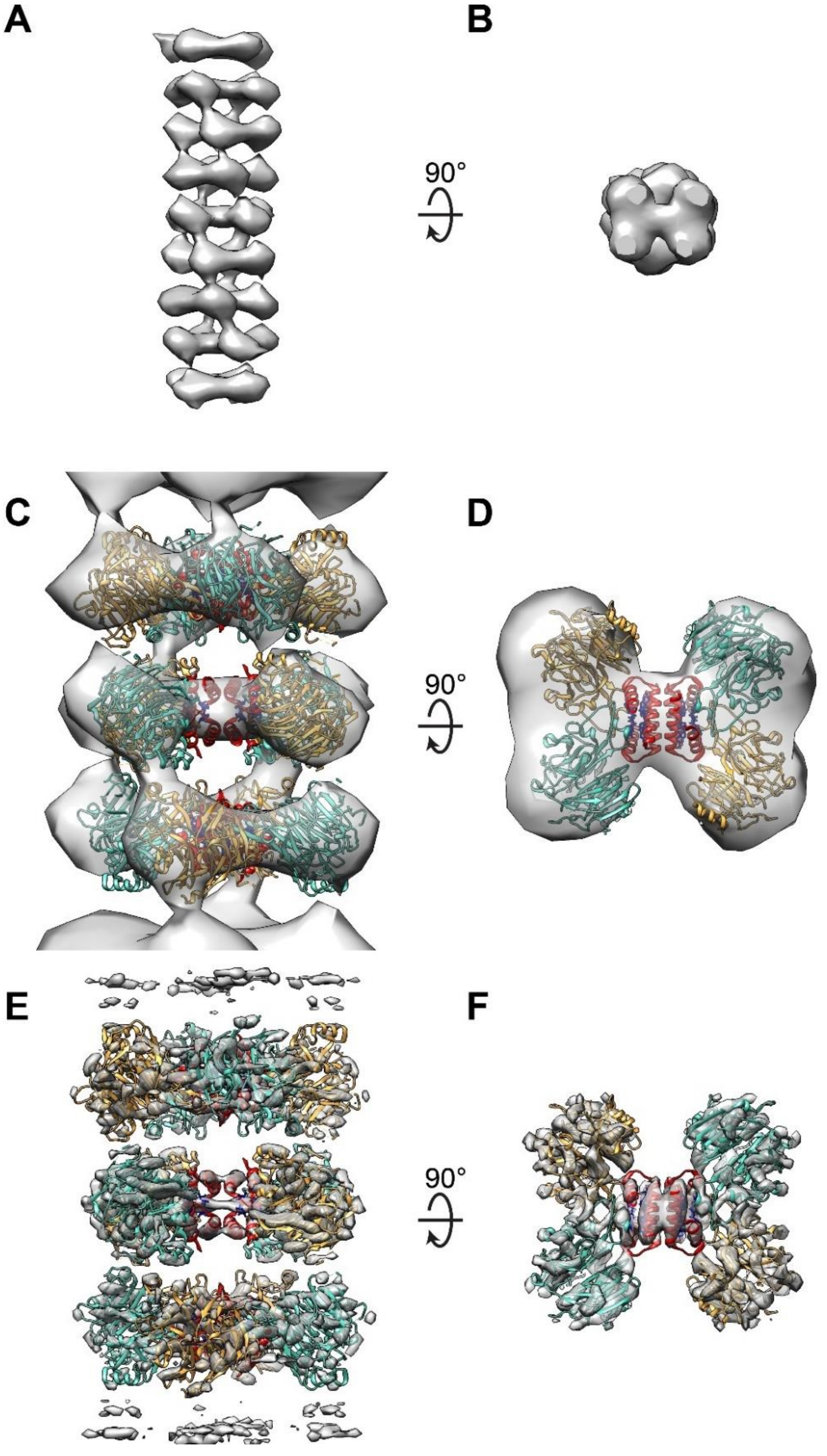

Supplementary Figure 3. Subtomogram averaging reconstructions of the RJ filaments. (**A** and **B**) *Ab initio* reconstruction obtained from subtomograms extracted from an individual RJ filament shown as surface representation. (**A**) Side view, (**B**) cross section along the longitudinal axis. (**C** and **D**) As in (A and B) with 3 copies of the crystal structure of the planar $MRJP_4$-$apimisin_4$ hetero-octamer (PDB ID 5YYL) fitted as rigid-body. (**E** and **F**) As in (C and D) showing the EM density of the final sub-nanometer tomographic reconstruction. The MRJP1 subunits in the hetero-octamer are alternately colored in orange and cyan to distinguish subunits from different hetero-octamers. Apisimin is colored in red, 24-methylenecholesterol in blue.

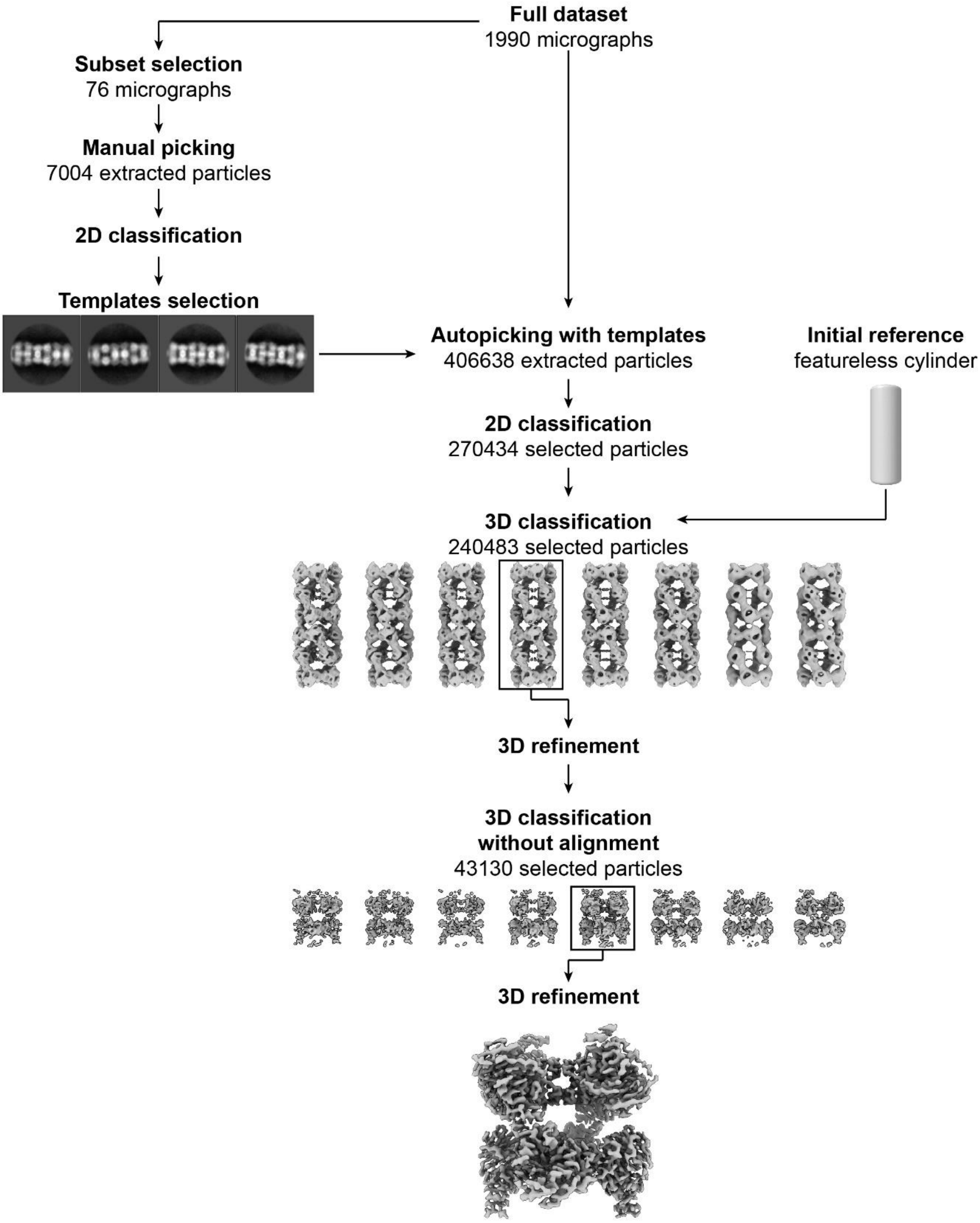

Supplementary Figure 4. Cryo-EM data processing and helical reconstruction of the RJ filaments. Processing scheme of the native RJ filaments sample. Manually picked particles were used to generate four 2D class averages for template-based autopicking. 2D classification was used to clean the dataset, 66% of the extracted particles were retained for further processing. A featureless cylinder was used as initial reference for 3D classification. After 3D classification, one class containing 89% of the particles was selected for 3D refinement and a further round of 3D classification without alignment. One class containing 18% of the particles was selected for 3D refinement that resulted in a final reconstruction at 3.5 Å resolution.

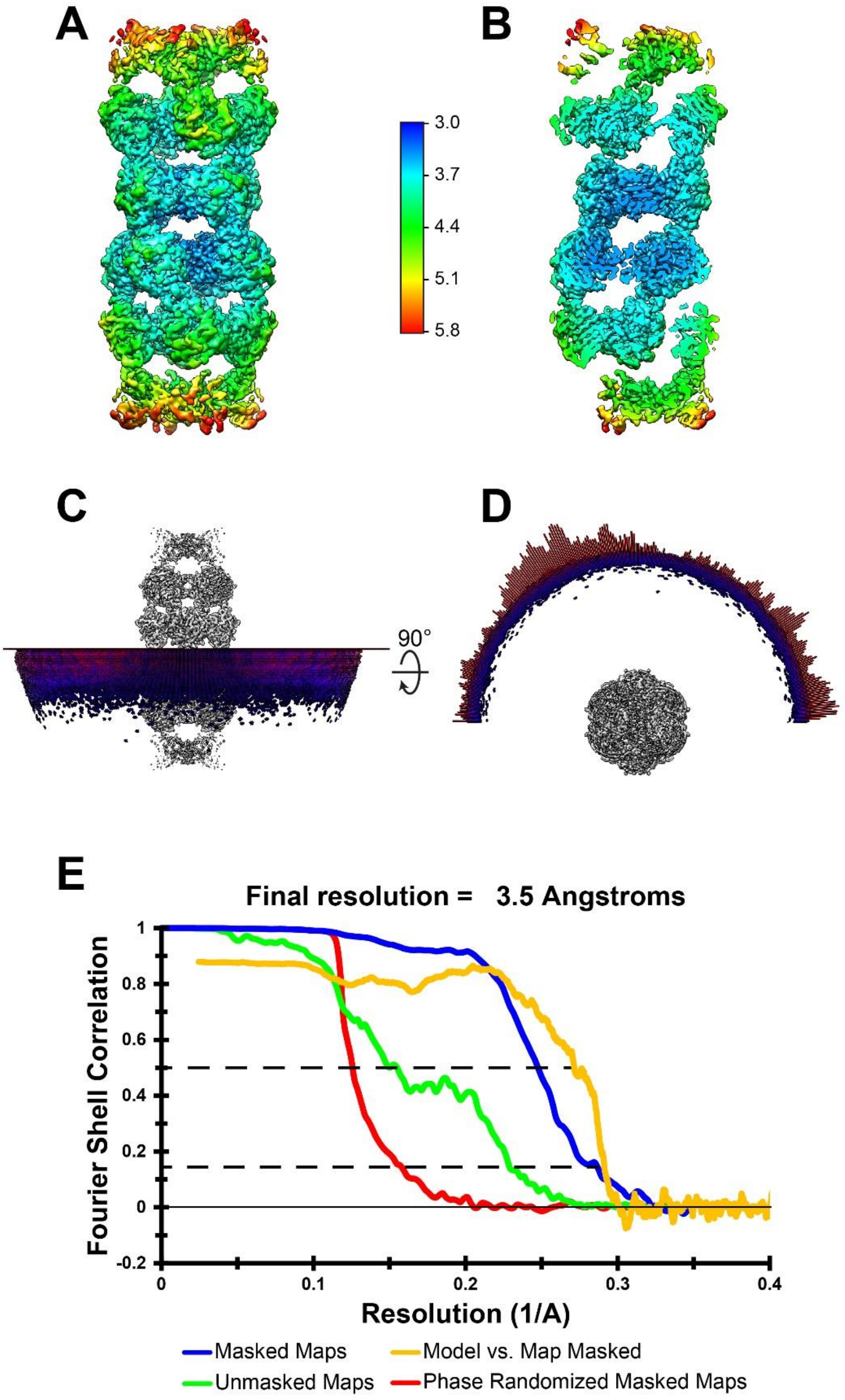

Supplementary Figure 5. Data quality and local resolution of helical reconstruction. (**A**) Local resolution map of the RJ helical assembly shown from a side view. (**B**) Cross-section along the longitudinal axis of the map shown in (A). The local resolution maps shown in (A) and (B) are colored according to the same color key. (**C** and **D**) Euler distribution plots are shown both from the side view (C) and from the top view (D). (**E**) Fourier shell correlation (FCS) curves of the helical reconstruction map. The overall resolution is estimated using the FSC = 0.143 criterion. The model-vs-map FSC curve shown in yellow reveals a similar resolution using an FSC = 0.5 cutoff.

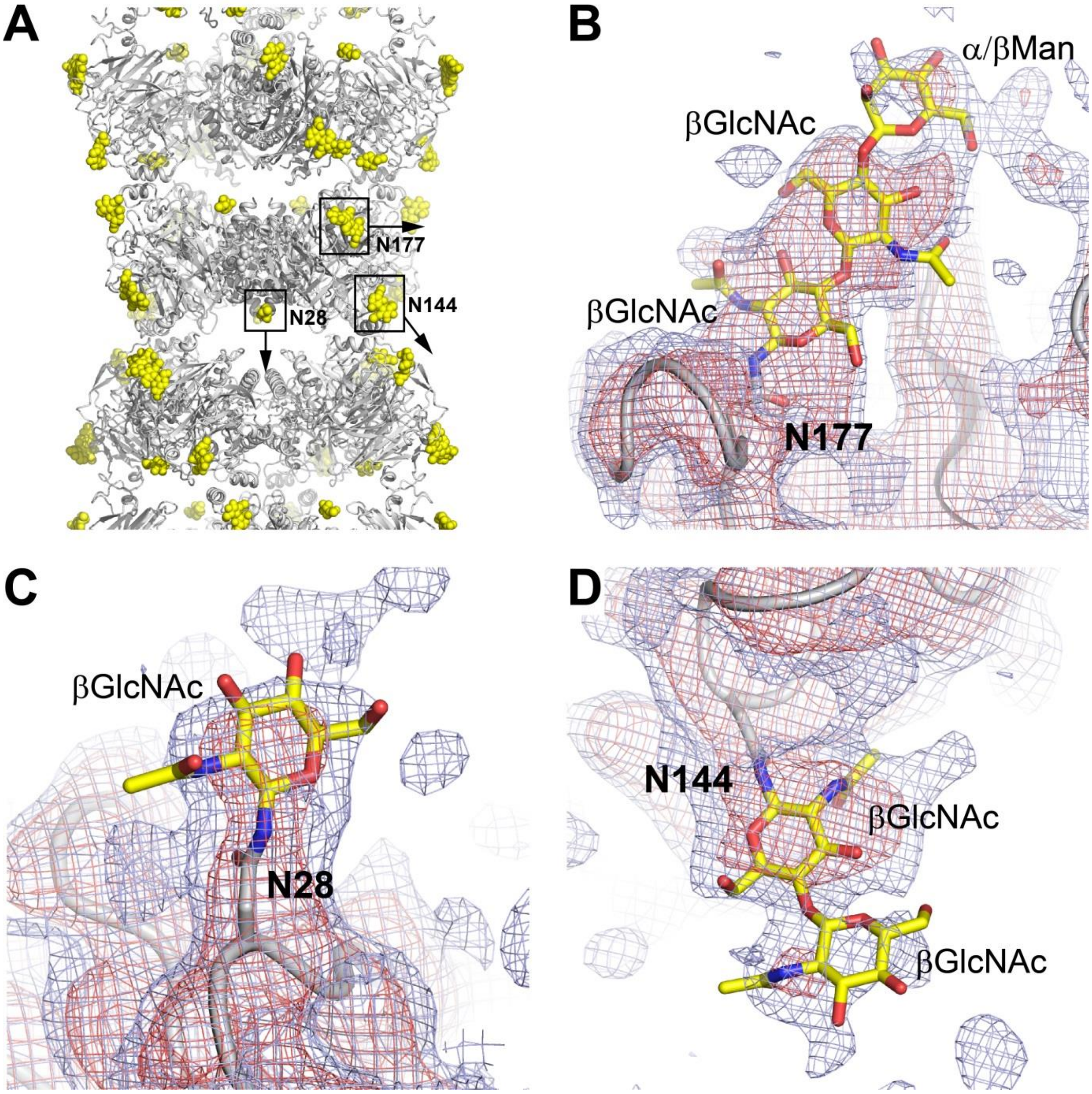

Supplementary Figure 6. N-glycosylation sites of native MRJP1 in native RJ filaments. (**A**) Overview of the N-glycosylation sites located at the solvent exposed surfaces of the RJ filament, with the attached sugars shown as yellow spheres and the modified asparagine residues labelled for one MRJP1 subunit. (**B**-**D**) Detailed views of the N-glycosylation sites. The EM density (contoured at two levels as red and blue mesh, respectively) unambiguously reveals sugar modifications at all three predicted sites, albeit it fades away from the asparagine attachment points (glycan conformations are not resolved at atomic resolution). Consequently, only some of the closest sugar moieties belonging to the canonical N-glycosylation pattern were tentatively modelled.

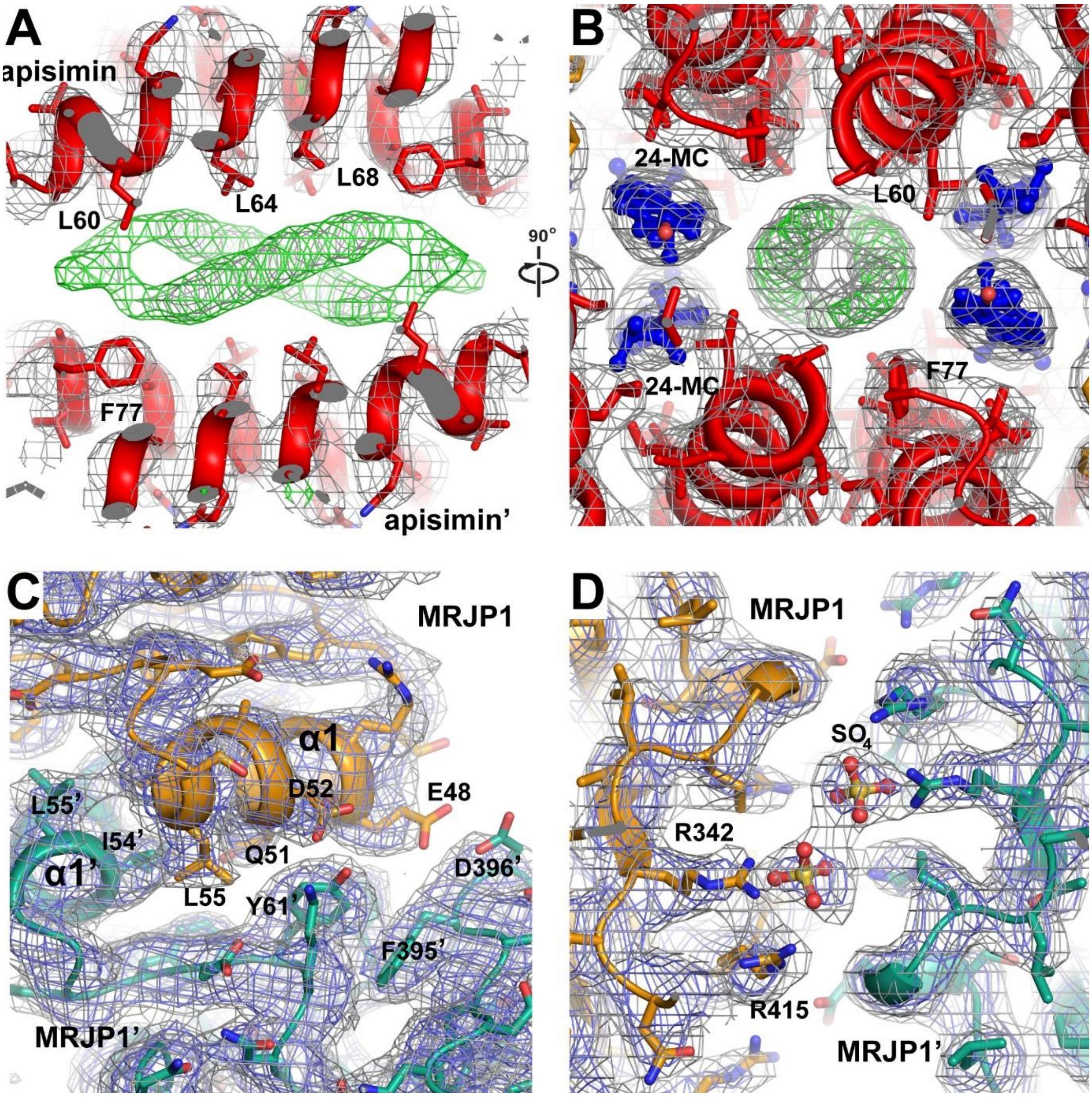

Supplementary Figure 7. Detailed views of EM densities discussed in the text. (**A, B**) Additional rod-shaped density is present in the core of each hetero-octamer, surrounded by hydrophobic apisimin residues (red) and 24-methylenecholesterol (24-MC, blue) molecules. EM difference and experimental EM densities, reminiscent of aliphatic tails of fatty acids, are shown as green and grey mesh, respectively. (**C**) Detailed view of the major contact area formed by alpha helix 1 of MRJP1 between neighboring layers in the RJ filament. EM densities are shown at two different contour levels as grey and slate mesh, respectively. (**D**) Sulfate ions bound at the periphery between symmetry related MRJP1 subunits within each layer in the RJ filament. The densities are shown as in (C).

Supplementary Table 1. Cryo-EM data collection, map refinement, model refinement and validation statistics of the native RJ helical assemblies.	
EMDB accession / PDB code	EMDB-11892/7ASD
**Data collection:**	
Microscope	FEI Titan Krios
Detector	Quantum K3
Voltage (keV)	300
Electron exposure (e^-/Å^2)	82
Pixel size (Å) (super-resolution mode)	0.42
Magnification (nominal)	105,000x
Defocus range (µm)	-1 – -3
Automation software	SerialEM
Selected micrographs	1990
**EM Reconstruction:**	
Initial particle images (no.)	406638
Final particles (no.)	43130
Accuracy of rotations (degrees)	0.64
Accuracy of translations (Å)	0.36
Resolution (masked) at FSC=0.143 (Å)	3.5
Sharpening B-factor (Å^2)	-96.6
**Coordinate real space refinement (PHENIX version 1.18.2-3874)**	
Unit cell	
a, b, c (Å)	149.33, 151.72, 127.83
$\alpha = \beta = \gamma$ (°)	90
CC_{mask}	0.83
High resolution limit used for refinement (Å)	3.5
Resolution according to model vs. map FSC=0.5 (masked) criterions (Å)	3.7
Model composition:	
Peptide chains	16 (8 MRJP1/apisimin protomers in two hetero-octameric layers)
Total atoms	30296
Protein residues	3664
Ligands: BMA / 94R / NAG / SO_4	8 / 16 / 40 / 8
Average B-factors (min/max/mean):	
Protein	40 / 179/ 86
Ligand	48 / 181 / 108
**Model validation:**	
RMSD bonds (Å)	0.002
RMSD angles (°)	0.508
All-atom clashscore	5.16
EMRinger score	2.67
MolProbity score	1.57
Ramachandran statistics:	
Favored (%)	97.8
Allowed (%)	2.2
Outliers (%)	0
Rotamer outliers (%)	2.16
